# Recent Advances in Advanced Membrane Materials for Natural Gas Purification: A Review of Material Design and Separation Mechanisms

**DOI:** 10.3390/membranes15120377

**Published:** 2025-12-09

**Authors:** Qijie Fan, Rui Xiao, Cheng Yang, Meixuan Xin, Xia Zheng, Guangyong Zeng

**Affiliations:** 1 Sichuan Lianfa Natural Gas Co., Ltd., Chengdu 610043, China; ffqj123@163.com (Q.F.); xrabc@163.com (R.X.); ycyc928123@163.com (C.Y.); 2 College of Materials and Chemistry & Chemical Engineering, Chengdu University of Technology, Chengdu 610059, China; 2023020666@stu.cdut.edu.cn (M.X.); zhengxia@stu.cdut.edu.cn (X.Z.)

**Keywords:** membrane materials, membrane modification, natural gas purification, separation mechanisms

## Abstract

Natural gas plays a pivotal role in the global energy landscape under the dual challenges of energy transition and climate change. However, the impurities present within natural gas pose several disadvantages, including corrosion of transportation pipelines, toxicity, hydrate formation, and a reduction in the fuel’s calorific value. Membrane separation technology has been recognized as an ideal approach for natural gas purification owing to its advantages of low energy consumption, operational simplicity, and excellent separation performance. This review summarizes recent progress in the development of advanced membrane materials, including polymer bulk membranes, two-dimensional (2D) nanosheet membranes, mixed-matrix membranes (MMMs), surface-modified membranes, and carbon molecular sieve membranes (CMSMs). The fundamental separation mechanisms—such as solution-diffusion, molecular sieving, adsorption-selectivity, and competitive sorption and surface diffusion—are analyzed in detail. Moreover, the critical scientific questions and technological challenges in this field are discussed in depth. Finally, future research perspectives are proposed to guide the rational design and practical application of high-performance membranes for natural gas separation.

## 1. Introduction

Natural gas, as a low-cost and high-efficiency clean energy source, plays a crucial role in ensuring the sustainable global energy supply and mitigating greenhouse gas emissions. It primarily consists of the combustible component methane (CH_4_), which typically accounts for 70–95% of its composition. However, natural gas also contains substantial amounts of non-combustible impurities such as carbon dioxide (CO_2_), hydrogen sulfide (H_2_S), nitrogen (N_2_), and water vapor (H_2_O) [1]. Among these, CO_2_ and H_2_S are acidic gases that can easily form hydrosulfuric acid and carbonic acid upon contact with water. These impurities can corrode transportation pipelines, with H_2_S itself being toxic, and its combustion produces sulfur dioxide (SO_2_), a major contributor to acid rain [2]. Additionally, inert gases like N_2_ lower the purity of natural gas, reducing the calorific value of the fuel. Particularly, the presence of water vapor, when transported under high pressure and low temperature conditions, can form hydrate crystals with methane, ethane, and other hydrocarbons. This can block pipelines, valves, instruments, and other equipment, leading to a rapid increase in transport pressure, interruptions in gas flow, and potential equipment damage [3,4]. Therefore, purification is essential before natural gas can meet pipeline transportation standards. Consequently, the development of clean, efficient, and facile separation technologies has become an urgent necessity.

Commonly used natural gas purification techniques include amine absorption, physical solvent methods, membrane separation, cryogenic separation, and pressure swing adsorption [5,6,7,8]. Among these, membrane separation has emerged as one of the most promising approaches due to its remarkable advantages, including low energy consumption, cost-effectiveness, operational simplicity, modular design, and environmental friendliness [9,10,11]. It is particularly suitable for compact or space-limited applications [12]. In practical purification processes, membrane technology enables the simultaneous removal of multiple impurities from natural gas and exhibits excellent separation performance—both in permeability and selectivity—toward acidic gases such as H_2_S and CO_2_ [13,14]. However, conventional membrane materials rely on a single separation mechanism and are generally suitable only for mild purification conditions. Moreover, these membranes are constrained by the Robeson upper bound, which reflects the inherent trade-off between permeability and selectivity. They also suffer from limitations such as physical aging, susceptibility to blocking, and limited stability, making it difficult for them to meet the current pipeline transportation standards [15,16]. To overcome the limitations of conventional membranes, researchers have developed a variety of advanced high-performance membrane materials through chemical structure regulation and physical morphology design [17,18,19]. By employing strategies such as surface functionalization, internal structural optimization, and the incorporation of composite components, these membranes achieve a favorable balance between high permeability and enhanced selectivity [15,19]. Furthermore, they exhibit superior resistance to plasticization, improved anti-blocking performance, and excellent long-term operational stability, offering a promising pathway for efficient natural gas purification [17,20,21].

**Figure 1:**
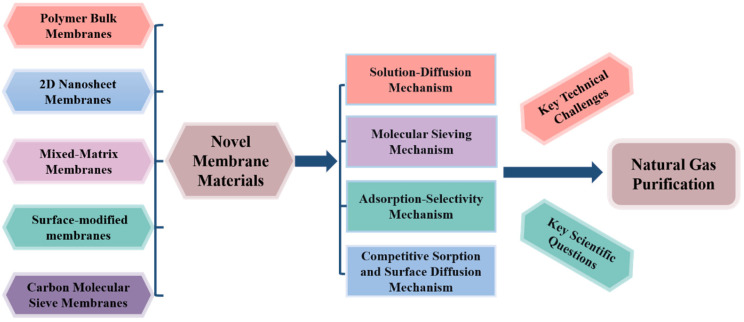
Schematic illustration of the overall framework and conceptual roadmap.

Therefore, this review provides an overview of recent advances in the design, fabrication, and modification of novel membrane materials for natural gas purification. The separation mechanisms of these membranes in removing impurity gases are analyzed in detail, and future research directions in this field are discussed. This work aims to provide theoretical insights and practical guidance for the development of low-carbon and efficient membrane-based gas purification technologies. The overall framework of this review is illustrated in Figure 1.

## 2. Gas Purification Efficiency of Membrane Technologies and Other Separation Methods

### 2.1. Efficiency of Membrane Separation Technology

Membrane separation technology offers several intrinsic advantages, including low energy consumption, modular equipment design, simple operation, and strong adaptability to compact or space-limited installations. Its separation efficiency is fundamentally governed by the membrane material’s permeability and selectivity [22]. Permeability reflects the amount of gas that can pass through a unit area of membrane per unit time, representing the processing throughput, whereas selectivity describes the membrane’s ability to discriminate between different gas species, which directly determines product purity and recovery [23]. The trade-off between these two parameters defines the overall purification potential of membrane materials in natural gas treatment [24].

Emerging membrane materials—compared with conventional polymeric membranes such as polyimides or cellulose acetates—have focused on surpassing the Robeson limit, optimizing fabrication and operational conditions, and improving long-term stability [25]. These developments have led to multiple high-performance membranes capable of significantly enhancing natural gas purification efficiency. Presently, single-stage membrane systems, while effective at removing H_2_O, H_2_S, and CO_2_ despite the Robeson upper bound constraint, perform poorly in rejecting N_2_ and heavy hydrocarbons due to pore blockage by slow-permeating heavy hydrocarbons and the similar permeabilities of N_2_ and CH_4_ [26,27].

### 2.2. Other Gas Purification Technologies

In addition to membrane separation, several established techniques are widely used for natural gas purification, including amine absorption, pressure swing adsorption, and cryogenic separation [5]. Their comparative performance, advantages, and limitations are summarized in Table 1.

Amine absorption, one of the most mature and reliable industrial processes, removes acidic impurities such as H_2_S and CO_2_ through reversible chemical reactions between these species and organic amine solvents [28,29]. It offers excellent purification efficiency, high processing capacity, broad applicability, stable operation, and regenerable solvents [30]. However, this method requires large and complex equipment, involves high energy consumption for solvent regeneration, may cause corrosion, and generates secondary pollutants [31,32].

Pressure swing adsorption relies on differences in adsorption capacity of gas components on porous adsorbents under high pressure. Impurities such as H_2_O, CO_2_, and H_2_S are selectively adsorbed and subsequently desorbed by lowering the pressure [5,33]. Pressure swing adsorption enables deep removal of moisture and CO_2_, provides high product purity, relatively low energy consumption, and allows automated operation under mild conditions [34]. Nevertheless, its treatment capacity is restricted by the size of adsorption columns, and during regeneration, a portion of the adsorbed CH_4_ is inevitably lost, reducing overall recovery. Additionally, its performance is less satisfactory when treating high-sulfur natural gas [35].

Cryogenic separation exploits differences in boiling points among gas components [36]. By lowering the temperature, simultaneous removal of H_2_O and heavy hydrocarbons can be achieved with high efficiency [37]. This method becomes especially attractive for large-scale applications due to its process simplicity and environmental compatibility [38]. However, its ability to deeply remove H_2_O is limited, and the formation of solid hydrates at low temperatures can block pipelines and equipment [39]. Furthermore, the severe low-temperature and high-pressure operating conditions impose strict requirements on equipment materials, increasing capital investment [37].

## 3. Membrane Materials for Natural Gas Purification

Novel membranes for natural gas purification can be broadly categorized into polymer bulk membranes, 2D nanosheet membranes, surface-modified membranes, and carbon molecular sieve membranes. Owing to their unique physicochemical properties, each of these materials exhibits distinct advantages and significant development potential. Table 2 summarizes the separation performance of various membrane materials developed in recent years for the removal of impurities from natural gas, highlighting their effectiveness in purifying methane from common contaminants such as CO_2_, H_2_S, and H_2_O.

### 3.1. Polymer Bulk Membranes

Polymer bulk membranes are the most widely used materials in natural gas purification due to their low cost and ease of large-scale production [60]. According to their glass transition temperature (Tg), they can be categorized into rubbery and glassy polymers [13]. At the operating temperature, the polymer chains in rubbery membranes are in a thermally mobile state, providing excellent elasticity and gas permeability. Common examples include polydimethylsiloxane (PDMS) [61,62] and polyether block amide (Pebax) [48,63], whose separation mechanism is primarily governed by solubility selectivity. However, the flexible structure of rubbery polymers makes it difficult to achieve the required gas separation performance, and under high pressure, surface defects can lead to swelling, thereby significantly shortening membrane lifespan [64,65,66]. In contrast, glassy polymers such as cellulose acetate (CA), polysulfone (PSF), and polyimide (PI) are non-equilibrium supercooled liquids characterized by high rigidity, superior selectivity, and excellent thermal and chemical stability [67,68]. Their separation performance mainly depends on the intrinsic microporous structure between polymer chains, which is dominated by differences in diffusion coefficients, leading to outstanding selectivity [69]. However, these membranes generally possess a dense, non-porous internal structure, which results in inherently low permeability. In addition, they are prone to plasticization and pronounced physical aging when exposed to high-pressure operating conditions [70,71,72].

**Figure 2:**
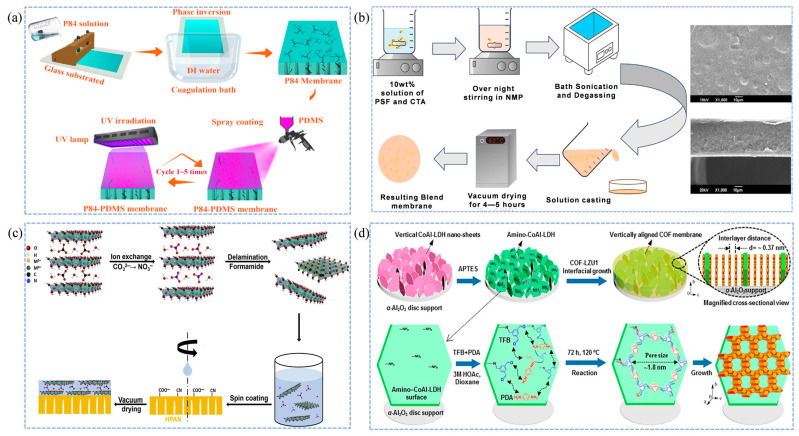
Schematic fabrication processes of membranes for natural gas purification. (**a**) Fabrication of P84-PDMS composite membranes [41]; (**b**) Fabrication of CTA/PSF membranes [43]; (**c**) Preparation of LDH membranes containing interlayer NO_3_^−^ [45]; (**d**) Preparation of 2D COF-LZU1 membranes [47].

Current research on polymer bulk membranes primarily focuses on functional modification, polymer innovation, and optimization of fabrication procedures, aiming to develop ideal polymeric membranes that simultaneously exhibit high permeability, strong selectivity, and outstanding operational stability [73]. Among them, polyimides have been widely engineered by introducing bulky pendant groups or applying crosslinking strategies, both of which significantly enhance their position relative to the Robeson upper bound and improve resistance to physical aging [74]. Polymer composite membrane, which integrate rubbery and glassy polymers in a well-designed configuration, further address the limitations of single-component polymer membranes, such as inadequate separation performance, physical aging, and poor long-term stability, while maintaining high cost-effectiveness [75]. Suleman et al. [40] fabricated PSF/PDMS composite membranes via a dip-coating process and systematically investigated the effects of operating pressure and blending ratio. Compared with pristine PSF membranes, the PSF/PDMS composites exhibited simultaneous enhancements in both permeability and selectivity. Notably, the permeability difference between CO_2_ and CH_4_ increased with rising pressure, while the CO_2_/CH_4_ selectivity improved significantly from 19.2 to 56.7. Building on this idea of functional enhancement, Li et al. [41] further explored membrane repair strategies by employing a rapid layer-by-layer assembly approach via PDMS spraying to restore copolyimide (P84) membranes. Under UV irradiation, the polymer membranes were able to self-heal within an ultrashort time (20–30 s), and their results showed that the P84-PDMS-3A membrane, prepared using a triple PDMS spraying concentration and two deposition cycles, delivered the best gas-separation performance. Compared with the original P84 membrane, the increased coating thickness reduced the permeability of larger molecules (CO_2_ and CH_4_), thereby widening the permeability gap between H_2_ and CH_4_ and resulting in a 2.4-fold enhancement in H_2_/CH_4_ selectivity, reaching as high as 231.9. The fabrication process is illustrated in Figure 2a. Roafi et al. [43] also successfully combined cellulose triacetate (CTA) and PSF polymer membranes. As illustrated in Figure 2b, their fabrication method yielded a dense, defect-free CTA/PSF membrane, which exhibited significantly enhanced selectivity without compromising permeability compared to the original CTA and PSF membranes, demonstrating great potential for CO_2_ removal.

### 3.2. Two-Dimensional Nanosheet Membranes

Two-dimensional (2D) nanosheet membranes, including graphene oxide (GO) [44,76], MXene [77], 2D metal–organic frameworks (MOFs) [78], and layered double hydroxides (LDHs) [45], have attracted considerable attention due to their unique 2D nanoporous structures and tunable properties [79]. These membranes provide promising solutions to the limitations of conventional polymer membranes, such as low permeation flux, the Robeson upper bound, and suboptimal separation performance [80]. The precise control of interlayer spacing and surface functional groups of 2D nanosheets—typically achieved through physical intercalation, chemical etching, or related strategies—is crucial for constructing 2D nanosheet membranes, which enables the selective separation of different gas molecules [81]. For example, Ren et al. [44] used vacuum filtration to filter GO modified with polydopamine (PDA) and Zn^2+^ onto a polyethersulfone (PES) substrate, successfully preparing GO-PDA-Zn^2+^@PES nanosheet membrane materials. The crosslinking interactions between Zn^2+^ and PDA not only expanded the interlayer spacing of the GO framework and elongated the internal nanochannel structures, but also effectively repaired surface defects on the GO membrane. Moreover, the presence of water molecules under humid conditions facilitated preferential CO_2_ transport, resulting in a significantly enhanced CO_2_/CH_4_ selectivity of up to 32.9. In addition, due to the strong affinity between CO_2_ and the membrane surface, the CO_2_ permeability increased markedly by approximately 61% compared with pristine GO membranes.

However, in practical industrial applications, 2D nanomaterials are costly and prone to issues such as interlayer swelling, structural aging, and membrane blocking under harsh operating conditions [82,83,84]. Liu et al. [45] prepared a smooth-surfaced LDH membrane using a co-precipitation-hydrothermal aging method and found that the intrinsic nitrate ions (NO_3_^−^) within the interlayers could undergo ion exchange with CO_2_, converting into carbonate ions (CO_3_^2−^). Owing to the high electronegativity and small size of CO_3_^2−^, the interlayer spacing of the LDH decreased from 0.7 nm to 0.3 nm, which effectively enhanced the separation performance. When the membrane thickness was increased to 70 nm, the CO_2_/CH_4_ selectivity improved from 33 to 37. However, the permeability of both CO_2_ and CH_4_ decreased, dropping to 105 and 1, respectively. Furthermore, the LDH membrane maintained a CO_2_/CH_4_ selectivity of approximately 30 during a 144 h operation test, demonstrating excellent separation performance and stability. The fabrication process is illustrated in Figure 2c. Fan et al. [47] designed a vertical COF-LZU1 membrane, with its fabrication process detailed in Figure 2d. In contrast to a pure COF 2D membrane, this configuration features a more refined internal structure, which is attributed to a substantial increase in permeability up to 3800 GPU while retaining a CO_2_/CH_4_ selectivity of 31.6.

### 3.3. Mixed-Matrix Membranes

Mixed-matrix membranes (MMMs) are composite materials formed by uniformly dispersing porous fillers with excellent molecular sieving performance or high permeability, such as zeolites [85,86], MOFs [87,88], polystyrene [89], and 2D materials [90], into a polymer matrix. The incorporation of these fillers generates well-ordered and tunable pore structures within the polymer membrane, while interactions between the fillers and polymer chains restrict chain mobility [51,91]. This combination significantly enhances both the permeability and selectivity of the membrane, surpassing the Robeson upper bound of conventional polymer membranes, and integrates the advantages of both the polymer matrix and the porous fillers [48,49,50,92].

**Figure 3:**
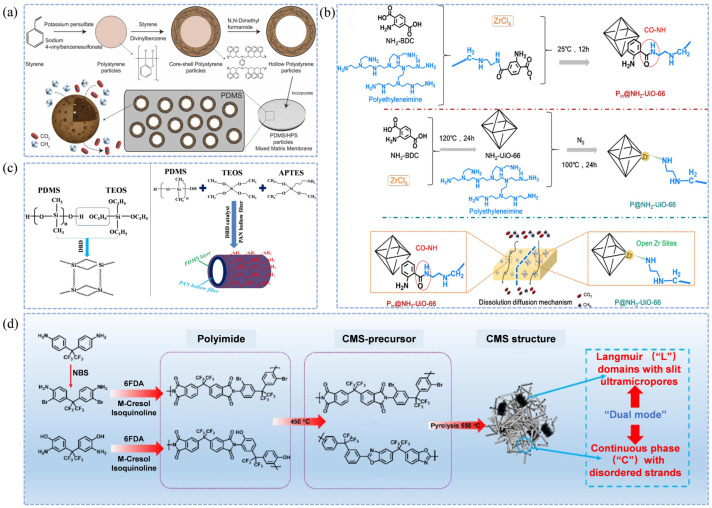
Schematic fabrication processes of membranes for natural gas purification. (**a**) Fabrication of PDMS/HPS MMMs [49]; (**b**) Fabrication of 30-Pin@NH_2_-UiO-66-PEI membranes [50]; (**c**) Fabrication of APTES/PDMS@PAN membranes [55]; (**d**) Thermal rearrangement and pyrolysis processes of PI-OH-550 and PI-Br-550 membranes [56].

The structure and performance of porous fillers are critical factors in the development of MMMs [93,94]. Martínez-Izquierdo et al. [48] successfully prepared ultrasmall UiO-66-NO_2_ particles (4–6 nm) and co-embedded them with ZIF-94 into a Pebax polymer matrix, resulting in UiO-66/Pebax 1657 thin-film nanocomposite membranes. The abundant surface amino, nitro, and hydrophilic groups on these fillers enhanced interactions with CO_2_, increased the membrane’s hydrophilicity, and promoted CO_2_ affinity, thereby achieving improved CO_2_/CH_4_ selectivity. The compatibility between porous fillers and the polymer matrix is also crucial. Unlike conventional inorganic fillers, Guo et al. [49] employed an improved template polymerization method to synthesize hollow polystyrene (HPS) particles, which were incorporated into a PDMS polymer matrix to fabricate PDMS/HPS MMMs. The results demonstrated that both PDMS and HPS being polymeric materials ensured excellent interfacial compatibility; SEM characterization revealed a smooth membrane interface without observable defect pores. Moreover, both the CO_2_/CH_4_ permeability and selectivity increased with the loading of HPS, reaching a maximum at 4 wt%, showing significant improvements compared with pristine PDMS membranes (259% and 37%, respectively). The CO_2_ permeability increased from 1.45 GPU to 5.21 GPU, while the selectivity rose to 5.86. The fabrication process is illustrated in Figure 3a. Cui et al. [50] fabricated a Pin@NH_2_-UiO-66-PEI mixed matrix membrane, with the preparation process detailed in Figure 3b. The amino groups on the MOF surface enhanced the membrane’s affinity for CO_2_ and expanded its internal porous structure. Consequently, CO_2_/CH_4_ permeability and selectivity were increased by 21 times and 4 times, respectively, compared to the pristine PEI membrane.

### 3.4. Surface-Modified Membranes

Enhancing the separation performance of membrane materials has always been a key issue in natural gas purification. Surface-modified membranes improve the interaction between the membrane material and acidic gas molecules through physical or chemical modification of the membrane surface [95,96,97]. These modifications are generally classified into two main strategies: surface grafting and surface coating [98,99]. Surface coating refers to the deposition of a functional layer onto the membrane substrate via physical or chemical means, such as dip-coating [100], spray-coating [101], or vapor deposition [102]. Aydani et al. [52] prepared Si-functionalized SSZ-13 membranes by dip-coating SSZ-13 molecular sieve membranes into a silica sol solution. Experimental results demonstrated that the modification effectively reduced surface defects. The membrane exhibited optimal performance at an ethanol/TEOS molar ratio of 95.5, achieving a CO_2_/CH_4_ selectivity as high as 660. This enhancement is attributed to the formation of siloxane (Si-O-Si) on the surface, which repaired the non-selective pores of the SSZ-13 membrane and improved the CO_2_/CH_4_ selectivity by more than fivefold. Similarly, Zhang et al. [53] employed a simple dip-coating approach to deposit an ultrathin Pebax/PEGDA-MXene selective layer on PVDF hollow fibers. The resulting layer exhibited a precisely controlled interlayer spacing of 3.59 Å—between the kinetic diameters of CH_4_ and CO_2_—achieving efficient CO_2_/CH_4_ separation. Furthermore, the attachment of PEGDA onto MXene enhanced the membrane’s affinity for CO_2_, resulting in a remarkable improvement in CO_2_/CH_4_ selectivity, reaching as high as 66.2. Moreover, after a long-term test of 160 h, the membrane maintained excellent separation performance, with selectivity remaining stable at approximately 68.

Plasma-induced grafting and chemical grafting are two common surface grafting techniques, differing mainly in the methods used to generate reactive sites such as free radicals [103]. In chemical grafting, these active sites are typically initiated by chemical reagents (e.g., peroxides or azo compounds) or by radiation sources (e.g., ultraviolet light or electron beams) [104,105]. The generated reactive species subsequently induce polymerization with monomers containing target functional groups (such as -NH_2_, -COOH, or epoxy groups), forming grafted polymer chains on the membrane surface. Huang et al. [54] employed plasma treatment under a helium atmosphere to induce chain scission in an intrinsic microporous polymer (PIM-1) membrane, thereby adjusting its hierarchical microporous structure. This modification enabled efficient CO_2_/CH_4_ separation, achieving a selectivity of 32.6—an increase of 165% compared with pristine PDMS membranes. Although the CO_2_ permeability decreased to 2045 GPU, it retained 82.6% of the original value, effectively enhancing the CO_2_/CH_4_ separation performance. Similarly, Hu et al. [55] utilized the strong affinity between amino groups and acidic gases to introduce amino-functionalized silane coupling agent (APTES) onto the PDMS surface through chemical grafting. Using a polyacrylonitrile (PAN) hollow fiber as the support, they fabricated an APTES/PDMS@PAN composite membrane with significantly improved surface polarity and gas separation performance. The abundance of nitrogen atoms on the membrane surface increased the surface polarity and hydrophilicity of PDMS. Moreover, with increasing APTES content, the crosslinked network became progressively more flexible, resulting in a continuous enhancement of CO_2_ permeability. This strategy effectively addressed the low permeability issue of nonpolar pristine PDMS membranes. The fabrication process is illustrated in Figure 3c.

### 3.5. Carbon Molecular Sieve Membranes

Carbon molecular sieve membranes (CMSMs), as typical intrinsic microporous membranes, are generally derived from polymer precursors such as phenolic resin [106], PI [107,108], or PAN [109] via high-temperature pyrolysis. These membranes possess rigid micropores with sizes around 3–5 Å, which enhance interactions with gas molecules [57]. Owing to the distinct kinetic diameters of different gas molecules and their varying interactions with the pore walls, CMSMs can achieve exceptional separation performance [110,111]. Consequently, these membranes enable the simultaneous removal of CO_2_, H_2_S, H_2_, and N_2_ impurities. CMSMs also exhibit excellent thermal and chemical stability, addressing plasticization issues of conventional polymer membranes under extreme conditions, extending membrane lifetime, and reducing secondary contamination, thereby offering significant potential for natural gas purification [112,113]. However, the precise control of pore size, together with the intrinsic brittleness of CMSMs, poses significant challenges for the fabrication of practical membrane modules. In addition, the high cost of suitable polymer precursors remains a major limitation for their large-scale application [114,115]. Sun et al. [56] designed two polymer precursors, PI-Br and debrominated PI-OH, which were thermally rearranged via pyrolysis to produce PI-OH-550 and PI-Br-550. Both membranes demonstrated excellent CO_2_/CH_4_ separation performance. Notably, the debrominated PI-Br-550 exhibited a dramatic increase in ultramicroporosity (from 10.4% to 83.03%), a reduced interlayer spacing, and abundant active sites. These structural advantages enabled exceptionally high CO_2_/CH_4_ and H_2_/CH_4_ selectivities of 45.5 and 102, respectively. Furthermore, high-temperature carbonization generated rigid aromatic structures within the membrane, increasing chain spacing and resulting in a remarkable enhancement in gas permeability—up to 199 times. The fabrication process is illustrated in Figure 3d.

## 4. Separation Mechanisms

The gas separation mechanisms of membrane technologies primarily include the solution-diffusion mechanism, the molecular sieving mechanism, and the adsorption-selectivity mechanism. In the field of natural gas purification, the separation behavior of membrane materials is often complex, with multiple mechanisms acting synergistically. Depending on the characteristics of the membrane, strategies such as surface modification, bulk functionalization, and hybrid material incorporation can be employed to achieve multi-mechanism cooperative separation, thereby enhancing the overall performance of natural gas purification membranes.

### 4.1. Solution-Diffusion Mechanism

**Figure 4:**
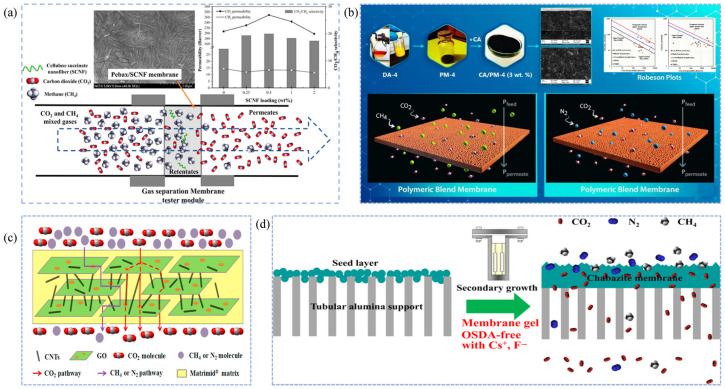
Schematic illustrations of gas separation mechanisms in natural gas purification membranes. (**a**,**b**) Solution-diffusion mechanism [127,128]; (**c**,**d**) Molecular sieving mechanism [129,130].

The solution-diffusion mechanism is the dominant separation mechanism for most dense membranes, such as polymeric membranes, and involves three consecutive steps: dissolution, diffusion, and desorption [116,117,118]. First, the gas impurities are adsorbed and dissolved in the membrane phase. This occurs because some membrane materials have a high free volume, and the solubility of gas molecules in the polymer membrane primarily depends on the critical temperature [119,120]. Polar molecules (such as H_2_O, H_2_S, CO_2_, etc.) tend to form hydrogen bonds or dipole interactions with the membrane and, having a higher critical temperature, are more likely to dissolve in the membrane phase [121]. Next, driven by the concentration gradient across the membrane, the gases diffuse to the other side of the membrane [122]. The rate of diffusion is determined primarily by the kinetic diameter and critical volume of the gas molecules. Finally, the gas species are desorbed from the membrane surface [123,124]. The core of this separation mechanism is the removal of polar gases, such as H_2_O, H_2_S, and CO_2_, from natural gas by exploiting the differences in solubility and diffusion coefficients of various gases [125,126]. For instance, Narkkun et al. [127] incorporated carboxyl-functionalized cellulose nanofibers (SCNF) into Pebax and fabricated Pebax/SCNF composite membranes via a solvent-casting method. Pebax provided abundant dissolution sites, while the SCNF regulated the microporous structure and enhanced CO_2_ solubility within the membrane, allowing CO_2_ to preferentially dissolve and diffuse through the membrane, resulting in efficient CO_2_/CH_4_ separation (Figure 4a). Akbarzadeh et al. [128] blended a glassy polymer (CA) with a rubbery polymer (PM-4) to prepare a high-performance polymer blend membrane (Figure 4b). Their study showed that the adsorption-dissolution sequence follows CO_2_ > CH_4_, and the presence of relatively large free-volume regions within the membrane promotes faster gas transport. This synergistic combination significantly improves both the permeability and selectivity compared with single-polymer membranes.

### 4.2. Molecular Sieving Mechanism

The core of the porous-membrane sieving mechanism lies in utilizing uniformly distributed and nanoscale pore channels within the membrane to separate natural-gas components based on differences in their molecular kinetic diameters [57,131]. The kinetic diameters of typical gas species are as follows: H_2_ (2.89 Å), H_2_O (2.65 Å), CO_2_ (3.30 Å), H_2_S (3.60 Å), N_2_ (3.64 Å), CH_2_ (3.80 Å), and heavy hydrocarbons (>3.80 Å) [129,132]. To efficiently remove acidic impurities such as CO_2_ and H_2_S from natural gas, the membrane pore size is typically tuned to fall between the kinetic diameters of CO_2_ and CH_4_ [133,134]. When high-pressure feed gas flows across the membrane surface, gas molecules are first selectively adsorbed onto the membrane and migrate toward the pore entrances. Subsequently, because the pore size is much smaller than the mean free path of the gas molecules, transport inside the membrane is dominated by Knudsen diffusion [110,135]. In this regime, frequent collisions between gas molecules and pore walls occur, enabling smaller CO_2_ molecules to permeate through the nanochannels toward the permeate side, while larger CH_4_ molecules are effectively hindered. Finally, desorption and gas collection occur on the low-pressure side of the membrane [136,137]. Therefore, this separation process is primarily governed by molecular sieving, and the precise design of pore size and internal structure is crucial for achieving high selectivity in natural-gas purification [138]. For example, Li et al. [129] developed a Matrimid@CNT/GO mixed-matrix membrane with both high selectivity and permeability. The incorporation of CNTs and GO nanosheets not only enhanced membrane permeability but also created internal pore channels that allowed CO_2_ passage while hindering CH_4_ transport, accelerating CO_2_/CH_4_ separation (Figure 4c). Liu et al. [130] fabricated CHA zeolite membranes on Al_2_O_3_ substrates by employing a secondary-growth method using an OSDA gel containing cesium and fluoride salts. This approach increased the thickness of the selective layer to approximately 6 µm, thereby enhancing the molecular-sieving performance of the membrane (Figure 4d).

### 4.3. Adsorption-Selectivity Mechanism

**Figure 5:**
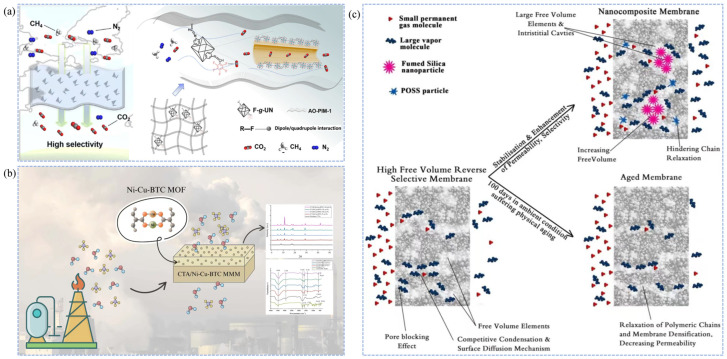
Schematic illustrations of gas separation mechanisms in natural gas purification membranes. (**a**,**b**) Adsorption-selectivity mechanism [148,149]; (**c**) Competitive sorption and surface diffusion mechanism [16].

The adsorption-selectivity mechanism is based on differential interactions between membrane materials and gas components (CH_4_, H_2_S, CO_2_, N_2_, etc.) on the membrane surface or within the pores [139,140]. The process involves adsorption, diffusion, and desorption steps, with adsorption serving as the core of the mechanism. Natural gas molecules are first adsorbed and dissolved on the membrane surface [141,142]. Subsequently, driven by the concentration gradient, they diffuse from the high-pressure side of the membrane to the low-pressure side, and are finally desorbed and collected [143,144]. Adsorption can be classified into physical and chemical types [145]. Physical adsorption depends on the membrane’s specific surface area, pore size, and surface polarity, whereas chemical adsorption is typically achieved by introducing functional groups that specifically interact with gas impurities, such as -NH_2_, -OH, -F, -COOH, or metal coordination sites [146,147]. Zhang et al. [148] grafted -F onto Zr-MOF particles to obtain F-g-UN nanoparticles, which were uniformly dispersed in AO-PIM-1 polymer membranes. The -F groups in the F-g-UN particles formed dipole-quadrupole interactions with CO_2_, selectively adsorbing CO_2_ and thus enhancing the CO_2_/CH_4_ separation performance (Figure 5a). Asad et al. [149] developed a novel mixed-matrix membrane by incorporating cellulose triacetate with bimetallic MOFs (Ni-Cu-BTC). The resulting membrane exhibited remarkable improvements in CO_2_ permeability and CO_2_/CH_4_ selectivity, increasing by 91.7% and 154.8%, respectively. These enhancements are attributed to the introduction of Ni-Cu-BTC, which generates abundant porous structures and active sites (unsaturated Ni^+^ and Cu^2+^) within the membrane, thereby increasing the gas diffusion coefficient and strengthening the adsorption affinity toward CO_2_, as illustrated in Figure 5b.

### 4.4. Competitive Sorption and Surface Diffusion Mechanism

Unlike the adsorption-selectivity mechanism, the competitive sorption and surface diffusion mechanism emphasizes the competition of various gas impurities for limited sites on the membrane surface [150,151]. Adsorption dominates this process, controlling the entire transport mechanism, followed by mass transfer separation via surface diffusion [150]. This mechanism is primarily found in glassy polymer membranes with ultra-high permeability, where selectivity is mainly determined by the different permeation rates of molecules through the polymer membrane [57]. An increase in permeability leads to enhanced selectivity [22]. In the case of natural gas components, polar gas molecules and heavier hydrocarbon components have higher condensation rates and critical temperatures compared to CH_4_ [152]. This means that impurity gases have higher permeability in ultra-permeable polymer membranes (such as glassy polymer membranes), thus achieving selectivity differences and purifying natural gas impurities [153]. Khosravi et al. [16] developed a high free-volume glassy polymer membrane, a novel PMP-FS-POSS mixed nanocomposite membrane for removing heavy hydrocarbon impurities. With the introduction of functionalized POSS-FS binary fillers, the free volume of the original polymer membrane increased, leading to higher permeability and strengthening the competitive sorption and surface diffusion mechanism, significantly improving the C_3_H_8_/CH_4_ separation performance (Figure 5c).

## 5. Key Scientific Questions and Technical Challenges

### 5.1. Key Scientific Questions

**Figure 6:**
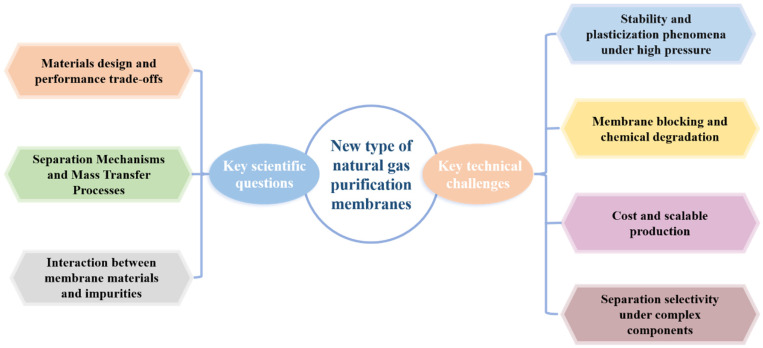
Schematic illustration of key scientific questions and technical challenges.

The application of membrane separation technology in natural gas purification involves several fundamental scientific questions. At its core, the challenge lies in understanding and optimizing the complex interactions among gas molecules, membrane materials, and operating environments. A summary of these key scientific questions and technical challenges is illustrated in Figure 6.

(1) Material design and the permeability-selectivity trade-off. A central scientific challenge in membrane science is to surpass the Robeson upper bound by designing membrane materials that combine high permeability with high selectivity. The separation performance of a membrane is closely related to its internal structure: larger pores enhance permeability but reduce selectivity, and vice versa [13]. Thus, precise molecular-level design of membrane microstructures is required. Strategies such as grafting or crosslinking rigid side groups (e.g., phenyl, heterocyclic, or conjugated rings) onto polymer backbones can finely tune the free volume distribution (0.3–0.5 nm) and improve gas diffusivity [154,155]. Alternatively, introducing specific functional groups (e.g., -NH_2_, -COOH, -OH, or metal centers) into polymer chains can enhance affinity toward acidic gases while suppressing CH_4_ permeation [156].

(2) Separation mechanisms and mass transport processes. Gas transport in advanced membranes follows multiple concurrent mechanisms, and the dominant mechanism varies with membrane type. For example, rubbery polymers primarily follow the solution–diffusion mechanism, governed by gas solubility differences [157]; glassy polymers, 2D membranes, MMMs, and CMSMs are mainly controlled by molecular sieving or surface diffusion [54,158]; while surface-modified membranes rely predominantly on adsorption-selective interactions due to the presence of reactive sites or polar functional groups [140]. Therefore, elucidating the adsorption sites, diffusion pathways, and competitive transport behaviors of CO_2_, H_2_S, and CH_4_ within different membrane structures is critical for improving separation efficiency.

(3) Interactions between membrane materials and gas impurities. Trace impurities in natural gas (e.g., CO_2_, H_2_O, heavy hydrocarbons, mercaptans) are not inert components—they can interact physically or chemically with membrane materials, leading to irreversible structural changes [159]. For instance, water molecules can induce hydrogen-bond reorganization and swelling in polymers [160]; high-pressure CO_2_ can plasticize glassy polymers, weaken interchain forces, and reduce selectivity [161]; and the acidity of H_2_S may hydrolyze certain polymer chains (e.g., cellulose acetate) or corrode inorganic fillers [162]. Unraveling these interaction mechanisms is essential for developing anti-aging, blocking-resistant, and long-term stable membranes for industrial natural gas purification.

### 5.2. Key Technical Challenges

Despite the promising prospects of membrane technology, its large-scale industrial application in natural gas purification still faces several significant engineering and technical challenges.

(1) Stability and plasticization under high pressure. Wellhead natural gas typically exists at pressures of several hundred to over a thousand psi. Under such conditions, nearly all polymer membranes experience permeability decay, with CO_2_-induced plasticization being particularly problematic [161]. Plasticization causes polymer chains to swell and lose selectivity, severely limiting long-term performance. Developing structurally stable membranes capable of maintaining separation efficiency under high pressure—such as rigid CMSMs or MMMs reinforced with rigid fillers—represents a critical research direction.

(2) Membrane blocking and chemical degradation. Feed gases often contain water vapor, heavy hydrocarbons, liquid droplets, and solid particulates, which can condense or deposit on the membrane surface, forming fouling layers that block pores and reduce permeability. Moreover, the coexistence of H_2_S, O_2_, and H_2_O can trigger oxidation and acid–base reactions, leading to chemical degradation of the membrane material and deterioration of performance [163]. Therefore, membrane materials must possess anti-blocking and corrosion-resistant properties, and effective, low-cost pretreatment processes should be developed to minimize contamination and degradation.

(3) Cost and scalable fabrication. Many high-performance membranes suffer from complex synthesis routes, harsh fabrication conditions, and high production costs, making large-scale manufacture challenging. For example, MOF membranes involve multi-step synthesis and delicate crystal growth control [164]; CMSMs require high-temperature pyrolysis with low yield [165]; while MMMs and 2D membranes often suffer from poor reproducibility and difficult process control [166]. Achieving cost-effective, high-throughput, and continuous membrane fabrication with consistent quality is therefore a crucial step toward commercialization.

(4) Selectivity under complex gas compositions. In practical applications, natural gas streams contain multiple impurities such as CO_2_, H_2_S, H_2_O, N_2_, and heavy hydrocarbons [167]. These components can interact competitively within the membrane, leading to decreased selectivity and separation efficiency [162,163]. Designing membranes capable of maintaining high selectivity toward specific target impurities (e.g., H_2_S or CO_2_) under multicomponent conditions remains a persistent and complex challenge.

## 6. Conclusions and Outlook

### 6.1. Conclusions

As a clean, efficient, and straightforward separation method, membrane separation technology has been widely applied in the field of natural gas purification due to its excellent gas separation performance and promising development potential. This review summarized the design principles and research progress of various novel membrane materials—including polymer bulk membranes, MMMs, 2D nanosheet membranes, and surface-modified membranes. The major separation mechanisms of these membranes in natural gas purification, such as the solution–diffusion mechanism, molecular sieving through porous structures, and adsorption–selectivity mechanism, were analyzed in detail. Furthermore, the fundamental scientific challenges—such as the trade-off between permeability and selectivity, complex mass transfer mechanisms, and membrane–component interactions—were discussed. On this basis, key technical barriers including high-pressure stability, membrane blocking, fabrication cost, and selective separation under multicomponent gas mixtures were also examined.

### 6.2. Outlook

Although membrane technology has shown great potential in natural gas purification, several challenges remain—particularly concerning long-term stability, anti-blocking performance, and large-scale fabrication cost. Future research directions can be as follows.

(1) Development of new high-performance materials. Explore novel membrane materials such as porous organic frameworks, advanced 2D materials, and bio-based membranes with enhanced selectivity and stability.

(2) Optimization of membrane structure design. Construct asymmetric or composite hollow-fiber membranes to improve gas permeance, mechanical strength, and operational durability.

(3) Process integration and intelligent control. Integrate membrane separation with adsorption or absorption processes to achieve synergistic effects, while incorporating intelligent monitoring and control strategies for enhanced process efficiency.

(4) Green and sustainable development. Emphasize renewable raw materials, low-energy fabrication, and membrane recyclability to promote environmentally friendly and sustainable membrane technologies.

In summary, with continuous innovation in material design, structural optimization, and process integration, membrane technology is expected to play an increasingly significant role in the green purification of natural gas—contributing to energy efficiency, carbon reduction, and sustainable development in the energy sector.

## Figures and Tables

**Table 1:** Comparison of gas purification efficiency and characteristics of different separation technologies.

Technology	CH_4_ Recovery Efficiency	Core Advantages	Major Limitations
Membrane Separation	High separation efficiency for H_2_O, H_2_S, CO_2_, and H_2_	Simple equipment Modular design Small footprint Low energy consumption Easy operation Environmentally friendly	Physical aging Membrane blocking Robeson upper bound Limited long-term stability
Amine Absorption	Highly efficient and deep removal of H_2_S and CO_2_	Mature technology High purification efficiency Widely applied Regenerable solvent	Large equipment size High energy consumption Corrosion issues Chemical waste generation
Pressure Swing Adsorption	High removal efficiency for H_2_S and CO_2_	High product purity Low energy consumption Fully automated Mild operation conditions	Lower product recovery Small processing capacity Product purity fluctuates with pressure cycles
Cryogenic Separation	Effective removal of H_2_O and heavy hydrocarbons	Simultaneous removal of water and heavy hydrocarbons Efficient at large scale Environmentally friendly	Clogging devices Limited removal effect High material requirements Product loss

**Table 2:** Separation performance of various emerging membrane materials for removing impurities from natural gas.

Membrane Material		Condition	Gas Pair	Permeability (GPU)	Selectivity	Ref.
Polymer Bulk Membranes	PSF/PDMS	25 °C 2 bar	CO_2_/CH_4_	CO_2_: 50 CH_4_: 1.1	56.7	[40]
	P84-PDMS-3A	35 °C 1 bar	H_2_/CH_4_ CO_2_/CH_4_	H_2_: 20.85 CO_2_: 3.65 CH_4_: 0.09	180 40.56	[41]
	6FDA-DAM:DAP(2:1)	25 °C 3.8 bar	CO_2_/CH_4_	CO_2_: 713 CH_4_: 25.3	28.2	[42]
	CTA/PSF	25 °C 4 bar	CO_2_/CH_4_	H_2_: 12 CO_2_: 1.12 CH_4_: 0.036	30.7	[43]
Two-Dimensional Nanosheet Membranes	GO-PDA-Zn^2+^@PES	30 °C 1 bar	CO_2_/CH_4_	CO_2_: 44	32.9	[44]
	LDH	30 °C 2 bar	CO_2_/CH_4_	CO_2_: 105 CH_4_: 1	37	[45]
	[Cu_2_Br(IN)_2_]n MOF	25 °C 1 bar	H_2_/CH_4_	H_2_: 527.4 CH_4_: 0.1	293.5	[46]
	2D COF-LZU1	25 °C 1 bar	H_2_/CH_4_	H_2_: 3671.1 CH_4_: 116.1	31.6	[47]
Mixed-Matrix Membranes	UiO-66/Pebax 1657	25 °C 0.15 bar	CO_2_/CH_4_	CO_2_: 192	19	[48]
	PDMS/HPS	25 °C 0.1 bar	CO_2_/CH_4_	CO_2_: 5.21	5.86	[49]
	30-Pin@NH_2_-UiO-66-PEI	25 °C 1 bar	CO_2_/CH_4_	CO_2_: 2498.9 CH_4_: 90.4	27	[50]
	NTU-101-NH_2_/6DFA-DAM	25 °C 1.5 bar	CO_2_/CH_4_	CO_2_: 190	43.9	[51]
Surface-Modified Membranes	Si-functionalized SSZ-13	30 °C 2 bar	CO_2_/CH_4_	CO_2_: 2370	660	[52]
	Pebax/PEGDA-MXene@PVDF	25 °C 1 bar	CO_2_/CH_4_	CO_2_: 770	55.2	[53]
	PIM-1	40 °C 6 bar	CO_2_/CH_4_	CO_2_: 2045	32.6	[54]
	APTES/PDMS@PAN	30 °C 0.2 bar	CO_2_/CH_4_	CO_2_: 1986	3.3	[55]
Carbon Molecular Sieve Membranes	PI-Br-550	30 °C 2 bar	H_2_/CH_4_ CO_2_/CH_4_	H_2_: 5590.8 CO_4_: 2492.4 CH_4_: 5.48	102 45.5	[56]
	CMS-600	25 °C 2 bar	CO_2_/CH_4_	CO_2_: 112.5	125	[57]
	PIM-PI(900 °C)	35 °C 2 bar	N_2_/CH_4_ CO_2_/CH_4_	N_2_: 0.7 CO_2_: 30 CH_4_: 0.02	35 1472	[58]
	6FDBPI-550	25 °C 2 bar	H_2_/CH_4_ CO_2_/CH_4_	H_2_: 3867.9 CO_2_: 2591.4 CH_4_: 83	46.6 37.1	[59]

## Data Availability

Not applicable.
